# Mapping a region within the 1RS.1BL translocation in common wheat affecting grain yield and canopy water status

**DOI:** 10.1007/s00122-014-2408-6

**Published:** 2014-10-18

**Authors:** Tyson Howell, Iago Hale, Ljupcho Jankuloski, Marcos Bonafede, Matthew Gilbert, Jorge Dubcovsky

**Affiliations:** 1 Department of Plant Sciences, University of California, Davis, CA 95616 USA; 2 Department of Biological Sciences, University of New Hampshire, Durham, NH 03824 USA; 3 Department of Genetics and Plant Breeding, Faculty of Agricultural Sciences and Food, 1000 Skopje, Macedonia; 4 Plant Breeding and Genetics Section, Joint FAO/IAEA Division of Nuclear Techniques in Food and Agriculture, IAEA, 1400 Vienna, Austria; 5 Instituto de Recursos Biológicos, CIRN, Instituto Nacional de Tecnología Agropecuaria (INTA), Buenos Aires, Argentina; 6 Howard Hughes Medical Institute, Chevy Chase, MD 20815 USA

## Abstract

**Key message:**

**This study identifies a small distal region of the 1RS chromosome from rye that has a positive impact on wheat yield.**

**Abstract:**

The translocation of the short arm of rye (*Secale cereale* L.) chromosome one (1RS) onto wheat (*Triticum aestivum* L.) chromosome 1B (1RS.1BL) is used in wheat breeding programs worldwide due to its positive effect on yield, particularly under abiotic stress. Unfortunately, this translocation is associated with poor bread-making quality. To mitigate this problem, the 1RS arm was engineered by the removal and replacement of two interstitial rye segments with wheat chromatin: a distal segment to introduce the *Glu*-*B3/Gli*-*B1* loci from wheat, and a proximal segment to remove the rye *Sec*-*1* locus. We used this engineered 1RS chromosome (henceforth 1RS^WW^) to develop and evaluate two sets of 1RS/1RS^WW^ near isogenic lines (NILs). Field trials showed that standard 1RS lines had significantly higher yield and better canopy water status than the 1RS^WW^ NILs in both well-watered and water-stressed environments. We intercrossed the 1RS and 1RS^WW^ lines and generated two additional NILs, one carrying the distal (1RS^RW^) and the other carrying the proximal (1RS^WR^) wheat segment. Lines not carrying the distal wheat region (1RS and 1RS^WR^) showed significant improvements in grain yield and canopy water status compared to NILs carrying the distal wheat segment (1RS^WW^ and 1RS^RW^), indicating that the 1RS region replaced by the distal wheat segment carries the beneficial allele(s). NILs without the distal wheat segment also showed higher carbon isotope discrimination and increased stomatal conductance, suggesting that these plants had improved access to water. The 1RS^WW^, 1RS^WR^ and 1RS^RW^ NILs have been deposited in the National Small Grains Collection.

**Electronic supplementary material:**

The online version of this article (doi:10.1007/s00122-014-2408-6) contains supplementary material, which is available to authorized users.

## Introduction

Wheat is an important food staple, estimated to account for approximately 20 % of all human food calories consumed worldwide (FAOSTAT 2014). Increases in wheat yields are required to accommodate rising food demand caused by the rapidly growing human population. One potential source for that improvement is the introgression of alien chromosomes, in particular the translocation of the short arm of rye (*Secale cereale* L.) chromosome one (1RS) into common wheat (*Triticum aestivum* L.) (Villareal et al. 1998; Owuoche et al. 2003; Ehdaie et al. 2011). This translocation was rapidly adopted worldwide because of the presence of resistance genes to several diseases and pests. Even after these resistance genes were overcome by new races of the different pathogens, the 1RS translocation has been retained in many wheat breeding programs (Graybosch 2001; Purnhauser et al. 2010) because of its contribution to increased yield potential (Kim and Johnson 2004), above ground biomass (Shearman et al. 2005), and better performance under abiotic stress, particularly drought stress (Villareal et al. 1991; Carver and Rayburn 1994; Schlegel and Meinel 1994; Moreno-Sevilla et al. 1995; Zarco-Hernandez et al. 2005; Hoffmann 2008; Ehdaie et al. 2011).

**Fig. 1:**
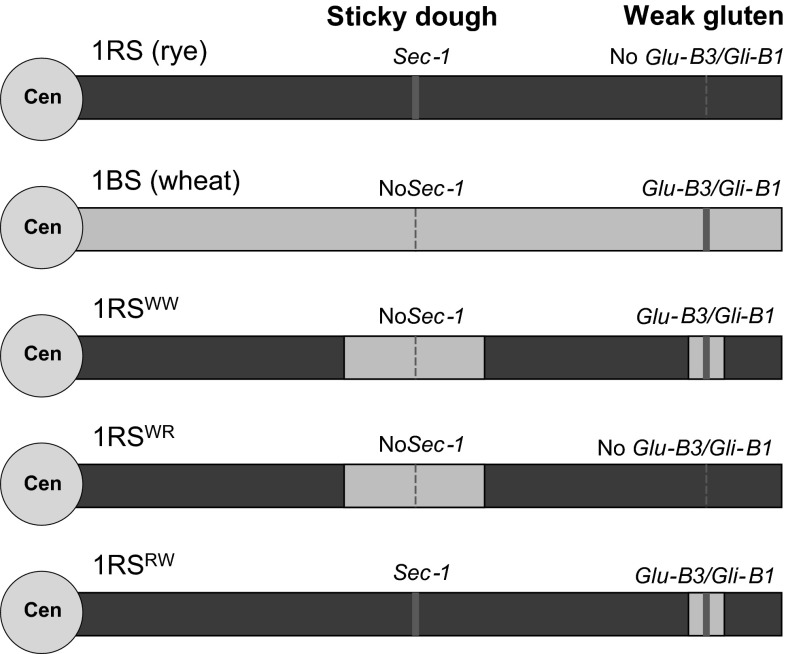
Chromatin configuration of each of the genotypes investigated; only the short arm of the chromosome is shown (all translocations have a complete wheat 1BL arm)

Unfortunately, breeding programs interested in bread-making quality have not been able to benefit from the 1RS.1BL translocation because of its undesirable associated traits for bread-making quality. The presence of the rye storage secalins encoded by the *Sec*-*1* locus, henceforth referred to as the proximal region, is associated with dough stickiness. The loss of the wheat low molecular weight glutenins (encoded by the *Glu*-*B3* locus) and possibly the linked gliadins (encoded by the *Gli*-*B1* locus), henceforth referred to as the distal region, is associated with reduced dough strength (Fenn et al. 1994). To address these quality concerns, a recombinant wheat-rye chromosome was engineered using induced homeologous recombination (Lukaszewski 2000). The newly engineered chromosome arm, henceforth referred to as 1RS^WW^ (for proximal and distal Wheat segments) has two short homeologous inserts of wheat chromatin: one replaces a proximal segment of rye chromatin with the *Sec*-*1* locus, and the other replaces a distal rye segment and introduces wheat storage protein loci *Glu*-*B3* and *Gli*-*B1* (Fig. 1). The wheat segments introgressed in the 1RS^WW^ line were selected using the closest available crossover points to the *Glu*-*B3/Gli*-*B1* and *Sec*-*1* loci (Lukaszewski 2000) to minimize the possibility of losing beneficial alleles from the rye chromatin.

Results from this study show that lines carrying the engineered 1RS^WW^ chromosome arm have significantly lower grain yields and canopy water status than near isogenic lines carrying the original 1RS chromosome arm. Although this is an unfortunate result for the direct utilization of the 1RS^WW^ chromosome arm in wheat breeding, it demonstrates that one or more genes responsible for higher grain yield and improved canopy water status are located within the 1RS chromatin replaced by one or both of the proximal and distal wheat segments introgressed. After separating the two introgressed wheat regions into pairs of independent near isogenic lines (NILs), we show that the region of the 1RS chromosome arm replaced by the distal wheat region is the one responsible for the improved water status and higher grain yield. Finally, we discuss the potential use of the germplasm generated in this study in wheat improvement.

## Materials and methods

### Plant materials

Two hexaploid wheat cultivars from the “Centro Internacional de Mejoramiento de Maíz y Trigo” (CIMMYT), cvs. ‘Attila’ and ‘Hahn’, were used as recurrent parents to study the effects of the 1RS^WW^ chromosome arm on grain yield and canopy water status. Both cultivars carry the same 1RS.1BL translocation used to generate the MA1 chromosome arm (Lukaszewski 2000), referred to in this study as 1RS^WW^. The 1RS chromosome arm carried by Attila, Hahn and cv. ‘Genaro’, which was the donor for the 1RS chromatin in the generation of the 1RS^WW^ chromosome arm, originated in diploid rye (2n = 14, genome RR) cv. ‘Petkus’, which was crossed to a German common wheat cultivar by Georg Riebesel in the 1950s (Rabinovich 1998) and eventually spread throughout the world through CIMMYT germplasm. In this study, Hahn and Attila were each crossed with a line containing the 1RS^WW^ chromosome arm in the cv. ‘Pavon 76’ background (kindly provided by Dr. Adam Lukaszewski), and backcrossed six times to the respective recurrent parent. The introgression of the 1RS^WW^ chromosome arm during the backcrossing process was monitored using A-PAGE. BC_6_F_1_ plants were self-pollinated and BC_6_F_2_ plants homozygous for 1RS^WW^ were selected. To confirm that the two 1RS^WW^ lines were nearly isogenic with their respective Hahn and Attila recurrent parents, all four lines were genotyped using the wheat Illumina 90,000 SNP iSelect array (Wang et al. 2014).

To separate the two wheat segments present in the 1RS^WW^ chromosome, the Hahn 1RS^WW^ line (PI 672837) was crossed to the original Hahn cultivar and two additional lines were generated: one carrying only the distal introgressed wheat segment, henceforth 1RS^RW^ (proximal Rye and distal Wheat, PI 672839), and one carrying only the proximal introgressed wheat segment, henceforth 1RS^WR^ (proximal Wheat and distal Rye, PI 672838) (Fig. 1). The Hahn background was selected to develop these lines because it showed larger differences between 1RS and 1RS^WW^ genotypes than Attila and Pavon 76. Ninety-five F_2_ plants were screened using molecular markers *o*-*sec*-*upp/o*-*sec*-*low* (Shimizu et al. 1997), a dominant proximal marker for the rye allele (primers described in Table 1), and *psp3000* (Devos et al. 1995), a dominant simple sequence repeat (SSR) marker completely linked to the *Glu*-*B3/Gli*-*B1* locus in wheat. Heterozygous individuals positive for both markers were selected for progeny tests, and two recombination events were detected. These two recombinants were used to generate lines homozygous for 1RS^RW^ or for 1RS^WR^. Two additional markers were later developed and were used to validate these recombinant lines. The first marker, created using sequence from the Diversity Arrays Technologies marker *wPt*-*1911* (Table 1), is a dominant marker for the wheat proximal region that complements the 1RS dominant marker *o*-*sec_upp*/*o*-*sec*-*low*. The second marker is a dominant SSR marker located within the distal wheat region, *gpw7059* (Table 1), which can also be used as a co-dominant marker by comparing the relative intensity of one of the amplified bands relative to the others.

**Table 1:** Molecular markers

Region	Allele	Primer name	Primer sequence
Proximal	Rye	*o*-*sec*-*upp* ^a^ *o*-*sec*-*low*	5′ TCA AGG TGT GTA GTG TAA AG 3′ 5′ GAC TTC ACT TCA TAA ATA GG 3′
Proximal	Wheat	*wPt*-*1911* F1^b^ *wPt*-*1911* R1	5′ GAG GTG TAC CGA TGG CTG TT 3′ 5′ GCA GTT GTG TAG CCG TTG TG 3′
Distal	Wheat	*psp3000* F1^c^ *psp3000* R1	5′ GCA GAC CTG TGT CAT TGG TC 3′ 5′ GAT ATA GTG GCA GCA GGA TAC G 3′
Distal	Wheat/rye	*gpw7059* F1^d^ *gpw7059* R1	5′ AAC ACC AAT GAC CTG ATC GC 3′ 5′ TCC TCA ACA GCT CCA GTG C 3′

^a^ Shimizu et al. (1997)

^b^ Diversity arrays technologies (www.diversityarrays.com)

^c^ Devos et al. (1995)

^d^ Sourdille and Bernard (unpublished data, http://wheat.pw.usda.gov/cgi-bin/graingenes/report.cgi?class=marker&name=GPW7059)

### Field experiments

Field experiments were conducted at the University of California field station at Davis, CA, (38°32′N, 121°46′W) and the Holtville, CA, Desert Research and Extension Center (DREC) (32°48′N, 115°26′W). Preliminary experiments were hand planted in 1-m rows (30 seeds per row) and subsequent small plot experiments were machine sown (2.97 million seeds per ha). Plot size averaged 4.5 m^2^ at Davis and 7.4 m^2^ at DREC. In Davis, sowing occurred in late October and early November (fall planting) in a Yolo loam soil. In DREC, the lines were sown in December (fall planting) in a Glenbar silty clay loam or clay loam soil. The Davis fields were sprayed with fungicide against stripe rust when needed; no fungicides were necessary at DREC. Insect control was not needed at any time at any of the locations. Experimental fields were flood irrigated when rain was not sufficient, except where irrigation was withheld for drought stress treatments.

One meter rows were hand harvested and small plots were harvested with a Wintersteiger Classic combine at maturity. Seed was cleaned and weighed to estimate grain yield. Temperature and precipitation data were obtained from the California Irrigation Management Information System website (http://www.cimis.water.ca.gov/). Planting and harvest dates, fertilization treatments, experimental design, and traits measured in each field experiment are summarized in Table 2.

**Table 2:** Experimental field design

Location	Year	Sowing date	Harvest date	Fertilization^a^ N	Genotypes^b^	Design and plot size^c^	Rep.^d^ (irr)	Traits^e^
Davis	08–09	10/30/08	6/11/09	67.25 kg/ha pre-plant and 2 × 44.8 kg/ha	H, H1RS^WW^, A, A1RS^WW^	RCBD split-plot 4.5 m^2^	10 (L)	Yield
Davis	10–11	11/09/10	7/5/11	67.25 kg/ha pre-plant and 2 × 44.8 kg/ha	H, H1RS^WW^, A, A1RS^WW^	RCBD split–split-plot 4.5 m^2^	6 (N × 2) 6 (L × 2)	Yield, CSR, CID
Davis	11–12	10/27/11	6/7/12	67.25 kg/ha pre-plant and 2 × 44.8 kg/ha	H, H1RS^WW^, H1RS^WR^, H1RS^RW^	RCBD 2 × 1 m row	20 (N)	CSR
Davis	12–13	11/6/12	6/5/13	67.25 kg/ha pre-plant and 2 × 44.8 kg/ha	H, H1RS^WW^, H1RS^WR^, H1RS^RW^	RCBD 4.5 m^2^	10 (N) 10(L)	Yield, CSR, GE, CID
DREC	09–10	12/12/09	5/20/10	112 kg/ha pre-plant and 135, 86 and 24 kg/ha	H, H1RS^WW^, A, A1RS^WW^	RCBD 2 × 1 m row	6 (N)	Yield, CID
DREC	10–11	12/15/10	5/5/11	134 kg/ha pre-plant and 136,100 kg/ha	H, H1RS^WW^, A, A1RS^WW^	RCBD split–split-plot 7.4 m^2^	6 (N) 6 (L)	Yield

^a^ N was applied as ammonium sulfate in Davis and anhydrous ammonia in DREC

^b^ *H* Hahn (1RS), *A* Attila (1RS)

^c^ In the split-plot designs, cultivar was used as the main plot factor and genotype (original 1RS vs 1RS^WW^) was used as the subplot factor. In split–split-plot designs, irrigation treatment was the main plot factor, cultivar was the subplot factor and genotype was the sub–subplot factor

^d^ *N* normal irrigation, *L* without final irrigation

^e^ *CSR* canopy spectral reflectance, *CID* carbon isotope discrimination, *GE* gas exchange

### Canopy spectral reflectance

Canopy spectral reflectance (CSR) data were collected using an Ocean Optics Jaz spectrometer (www.oceanoptics.com) with a low hydroxyl group (OH^−^) content fiber optic cable with no attached fore optic positioned approximately 50 cm above the top of the canopy in the nadir orientation. A poster board coated with a mixture of 60 % barium sulfate and 40 % white paint (w/w %) was used as a reference for 100 % reflectance. Measurements were taken between 9:30 am and 3:30 pm. During the 2010–2011 growing season, each recorded measurement was the average of 10 successive measurements of a fixed location within a plot, and three such measurements were taken per plot and later averaged (i.e., 30 subsamples per plot). In the following seasons, the measurement method was changed to a “scanning” method, where roughly 100 measurements were continually taken over the length of a plot and then averaged. The spectrometer model used generates canopy reflectance measurements approximately every 0.3–0.37 nm from 500 nm (green light) to 1000 nm (near infrared) wavelengths. Reflectance measurements from 10 adjacent wavelengths (approximately 3 nm) were averaged (boxcar smoothing parameter = 5) before calculating the water indices. The normalized water index 3 [NWI-3 = (R_970 nm_−R_880 nm_)/(R_970 nm_ + R_880 nm_)] (Gutierrez et al. 2010) was used to estimate the canopy water status, and the normalized difference vegetation index [NDVI = (R_900 nm_−R_680 nm_)/(R_900 nm_ + R_680 nm_)] (Rouse Rouse et al. 1974; Peñuelas et al. 1997) was used to estimate the canopy cover. For the 2011-2012 season, the original aluminum mirrors of the spectrometer were upgraded to silver mirrors and the grating was changed from Ocean Optics grating #4 (blaze wavelength 750 nm) to grating #14 (blaze wavelength 1000 nm) to optimize the measurement of wavelengths in the near infrared. NWI-3 field measurements made simultaneously with both spectrometer configurations were significantly correlated (*R* = 0.76, *P* < 0.0001), but the absolute values were offset due to the difference in light capture efficiency at 970 nm. A correction factor was calculated and used to adjust the NWI-3 values from the initial 2010–2011 field experiment at UC Davis to the same scale used in subsequent years, when the silver mirrors and the new grating were used.

### Carbon isotope discrimination

Carbon isotope discrimination (CID) was measured on whole grain flour from mature grains at the end of the season. Twenty-five grams of grains were ground for each sample in a UDY cyclone sample mill with a 1-mm screen. Samples were weighed into tin capsules and analyzed at the UC Davis Stable Isotope Facility (http://stableisotopefacility.ucdavis.edu/) for ^13^C/^12^C ratios. Isotope ratios were measured using a PDZ Europa ANCA-GSL elemental analyzer interfaced to a PDZ Europa 20-20 isotope ratio mass spectrometer (IRMS) (Sercon Ltd., Cheshire, UK). The resulting delta values (*δ*_p_) expressed relative to the international standard V-PDB (Vienna PeeDee Belemnite) were converted to CID (Δ) values using Eq. 6 from Farquhar et al. (1989) assuming a ^13^C/^12^C molar abundance ratio deviation of atmospheric CO_2_ (*δ*_a_) from the V-PDB standard of −8 per mil.

### Gas exchange

Gas exchange was measured on one sun-exposed flag leaf in each plot from the normal irrigation treatment on April 11, 2013, at the Davis site. A LI-COR6400XT gas exchange system with a 2 × 3 cm chamber and red-blue light sources was used for the measurements. Chamber conditions were: photosynthetic photon flux (PPF): 2000 μmol m^−2^ s^−1^, flow rate: 500 μmol s^−1^, and sample CO_2_: 400 μmol mol^−1^. The leaf temperature tracked ambient conditions and averaged 28.6 ± 3.8 °C (standard deviation). The high PPF resulted in light saturated conditions for photosynthesis and allowed a standardized estimate of the leaf photosynthetic capacity. The net photosynthetic rate and stomatal conductance to water were recorded less than 2 min after the leaf was placed in the chamber, once the photosynthetic rate stabilized. We confirmed that no changes in stomatal conductance occurred during the time needed for photosynthesis to stabilize. An external, shaded humidity and temperature sensor (HTM2500LF, Measurement Specialties, Inc., Hampton, VA 23666 United States) connected to the LI-COR was used to measure and record vapor pressure deficit (VPD). There was not a significant correlation between VPD and stomatal conductance, so VPD was not used as a covariable in the analysis of stomatal conductance.

Predicted CID values were calculated to assess the correlation between the gas exchange data from the leaves and the experimental CID measurements made on the grain. To do this, the intercellular CO_2_ value measured by gas exchange was used with the basic equation and CID values from Eq. 8 from Farquhar et al. (1989).

### Statistics

Statistical analyses were performed using SAS 9.3 (SAS Institute 2011). Analysis of variance (ANOVA) for yield, NWI-3, NDVI and CID data was performed using PROC MIXED. The variance components (VC) covariance structure was used to model a different variance component for each random effect. Experimental designs are summarized in Table 2. All means reported are adjusted least squares means (LS-means), and error bars are standard errors of the corresponding means. Differences among adjusted means were calculated using Tukey’s honestly significant difference (HSD) test at *α* = 0.05.

Photosynthetic rate was analyzed using ANCOVA with the logarithm of stomatal conductance as a covariable to account for possible environmental variation during the measurements, as described before (Gilbert et al. 2011). A highly significant regression (*R* = 0.71, *P* < 0.0001) was detected between these two variables, justifying the use of ANCOVA.

ANCOVA was used to correct yield data for a moisture gradient detected across the field in the DREC 2010–2011 experiment (Online Resource Fig. S1). Distance from the irrigation alley was used as covariable to account for this moisture gradient in both normal and low irrigation experiments.

In the Davis 2010–2011 experiment, full and limited irrigation treatments were replicated twice, allowing for the statistical testing between irrigation treatments. In the other experiments, irrigation treatments were not replicated due to logistical constraints, so differences between irrigation treatments or interactions between genotypes and irrigation treatments should be interpreted with caution.

## Results

### Comparison of 1RS and 1RS^WW^ genotypes

Analysis of the SNP data revealed that the Hahn and Attila parental lines were more closely related to each other than to the Pavon 76 line used as the donor parent of the wheat 1RS^WW^ chromosome arm. Excluding SNPs mapped to the 1B chromosome, which is involved in the 1RS.1BL translocation, 31,585 SNP markers were called in the Pavon 76 genotype. Of these SNPs, 5,810 (17.2 %) were polymorphic with the Hahn 1RS genotype and 5,812 (17.2 %) were polymorphic with the Attila 1RS genotype. After six generations of backcrossing to Hahn and Attila, the polymorphic Pavon 76 SNPs were reduced to 1 SNP (0.015 %) between the Hahn 1RS and 1RS^WW^ genotypes and 40 SNPs (0.6 %) between the Attila 1RS and 1RS^WW^ genotypes. These numbers are close to the 46 SNPs (0.8 %) expected after six backcrosses. Of the 33,734 SNPs called in the recurrent 1RS parents (including 1B SNPs), 231 (0.685 %) were polymorphic between the original Hahn 1RS and Attila 1RS genotypes and 307 (0.91 %) were polymorphic between the Hahn 1RS^WW^ and Attila 1RS^WW^ genotypes.

Overall, these results confirmed that the six backcross generations used to generate the lines used in this study resulted in highly isogenic 1RS and 1RS^WW^ pairs (slightly better in Hahn than in Attila). Therefore, the phenotypic differences observed between the 1RS and 1RS^WW^ lines within each cultivar have a high probability of being caused by the different wheat segments introgressed in the 1RS arm.

### Field performance evaluations of the 1RS and 1RS^WW^ NILs

#### Davis 2008–2009 preliminary experiment

**Fig. 2:**
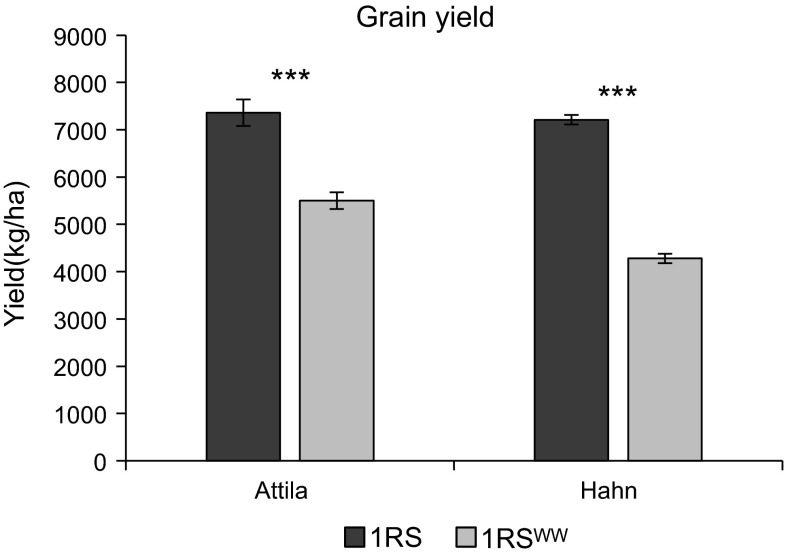
A Comparison of the adjusted mean yields between 1RS and 1RS^WW^ genotypes in the Hahn and Attila backgrounds (Davis 2008–2009)

The first field experiment comparing the original 1RS and the 1RS^WW^ chromosome arms in the Hahn and Attila backgrounds was performed at the UC Davis field site in 2008–2009. The split-plot design facilitated the visual comparison of the isogenic pairs, which showed visual differences in drought responses after 1 month without rain or irrigation between March 5th and April 6th 2009. In all ten replications and in both genetic backgrounds, the 1RS^WW^ genotype showed an increased proportion of rolled and dry leaves when compared to the respective recurrent parent with the original 1RS chromosome arm. Before water stress symptoms were evident in the NILs with the original 1RS chromosome arm, there was a series of late rains (38 mm) which limited further water stress. These particular conditions generated large yield differences between the 1RS and 1RS^WW^ NILs in both the Attila (1,857 kg/ha, 25.3 % decrease) and Hahn (2,928 kg/ha, 40.7 % decrease) cultivars (Fig. 2). The significant yield differences detected in both genetic backgrounds indicated that one or more valuable 1RS genes were missing in the 1RS^WW^ chromosome arm and motivated the initiation of this study.

#### DREC 2009–2010

**Fig. 3:**
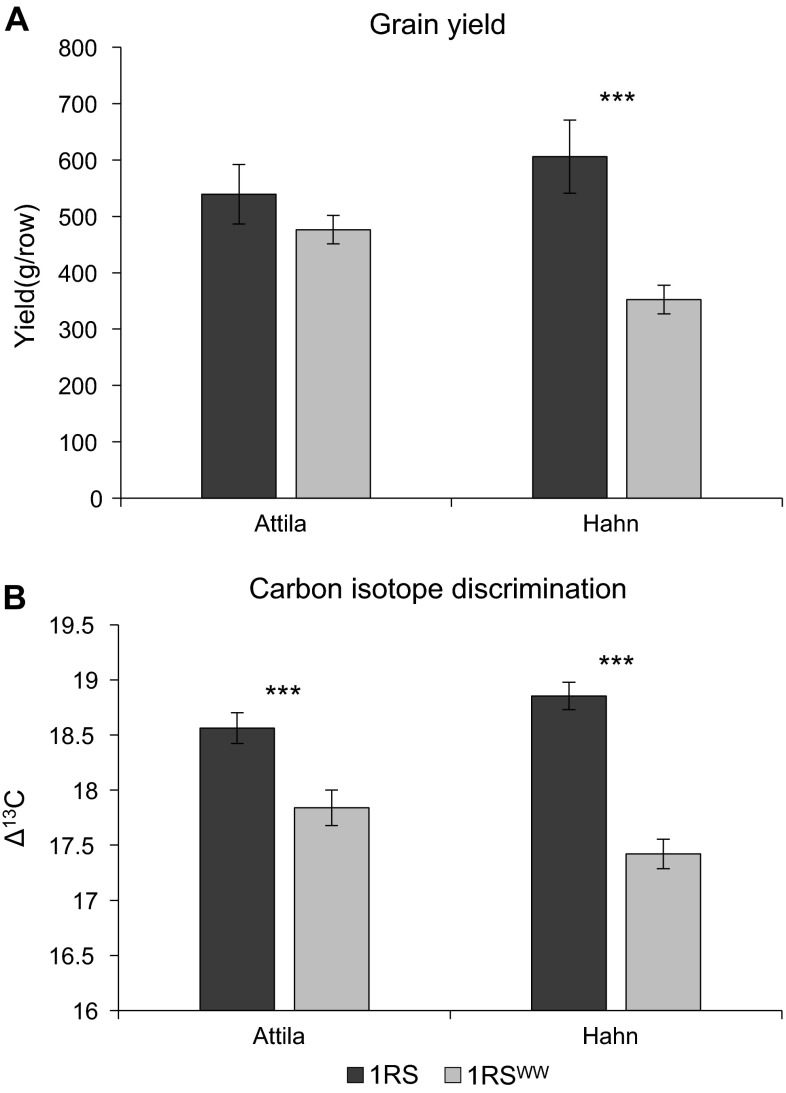
A comparison of the adjusted mean yields and CID values between 1RS and 1RS^WW^ genotypes in the Hahn and Attila backgrounds (DREC 2009–2010). **a** Adjusted mean yield comparison in grams per twin 1-m rows. **b** Adjusted mean carbon isotope discrimination values measured on flour from mature grains

In the next growing season, the same two pairs of NILs were compared in the Imperial Valley using two 1 m rows as experimental units. Although the main objective of this experiment was to measure CID, grain yield data were also collected. As in the Davis 2008–2009 experiment, the lines carrying the original 1RS chromosome arm had higher grain yield than those carrying the 1RS^WW^ chromosome arm, though the difference was significant only in the Hahn background (Fig. 3a). A significant interaction was observed between cultivar and 1RS-1RS^WW^ genotype (*P* = 0.0218, Fig. 3a).

Significant differences in CID were detected between the 1RS and 1RS^WW^ genotypes, with lines carrying the original 1RS chromosome arm showing higher discrimination than the respective NILs carrying the 1RS^WW^ chromosome arm (Fig. 3b). As in the yield data, the differences were larger in the Hahn NILs than in the Attila NILs and that was reflected in a significant interaction between cultivar and 1RS-1RS^WW^ genotype (*P* = 0.0005, Fig. 3b).

#### Davis 2010–2011 experiment

**Fig. 4:**
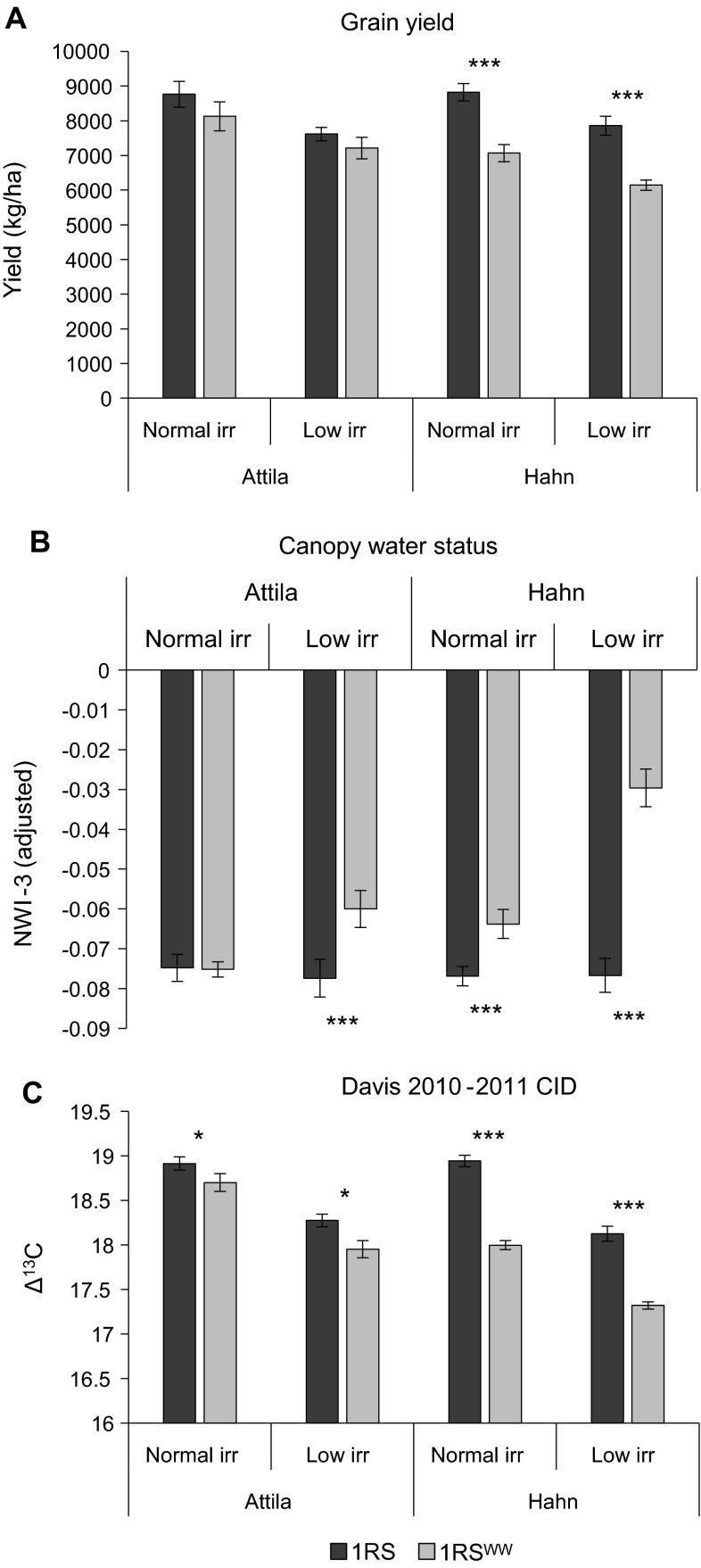
A comparison of the adjusted mean yields, NWI-3 and CID values between 1RS and 1RS^WW^ genotypes in the Hahn and Attila backgrounds under two different irrigation regimes (Davis 2010–2011). **a** Adjusted mean plot yield. **b** Adjusted mean canopy water status estimated by NWI-3 (more negative values indicate better canopy water status) as measured on May 6th and May 10th 2011. **c** Adjusted mean carbon isotope discrimination values measured on flour from mature grains

Based on the results from the two previous experiments, we organized a more complete experiment in Davis 2010–2011, including two areas with normal irrigation and two areas that received limited irrigation late in the season. As in the two previous experiments, the lines with the original 1RS chromosome arm had higher grain yield than the NILs with the 1RS^WW^ chromosome arm. The difference was significant only in the Hahn background (*P* < 0.0001, Fig. 4a), and a significant interaction was detected between genetic background and 1RS-1RS^WW^ genotypes (*P* = 0.0037). The interaction between irrigation treatment and the 1RS-1RS^WW^ genotype was not significant.

The analysis of NWI-3 based on the CSR data (Fig. 4b) also showed significant differences between 1RS-1RS^WW^ genotypes in both irrigation treatments for the Hahn NILs, but only in the limited irrigation treatment for Attila. The larger difference in the Hahn background was reflected in a significant interaction between the 1RS-1RS^WW^ genotype and cultivar (*P* < 0.0001). A significant interaction was also detected between genotype and irrigation treatment (*P* < 0.0001), with larger differences in NWI-3 values between NILs in the limited irrigation than in the full irrigation treatment, both for Attila and Hahn NILs (Fig. 4b).

The CID data showed the NILs with the original 1RS chromosome arm had significantly higher CID than the NILs with the 1RS^WW^ chromosome arm (Fig. 4c). A significant interaction was detected between the 1RS-1RS^WW^ genotype and cultivar (*P* < 0.0001), with larger differences between the Hahn NILs than between the Attila NILs. No significant interaction was detected between treatment and genotype for the CID values.

#### DREC 2010–2011 experiment

During the 2010-2011 growing season, a separate experiment was performed in the Imperial Valley in which the experiments with normal and reduced irrigation were located side-by-side, separated by a 6-m border with an irrigation alley located to the west of both treatments. A field “heat-map” of yield results showed an increase in yield with proximity to the irrigation alley in both treatments (Online Resource Fig. S1). To correct for this gradient, the distance to the irrigation alley was used as a covariable in an ANCOVA to correct for the yield gradient detected in both treatments.

**Fig. 5:**
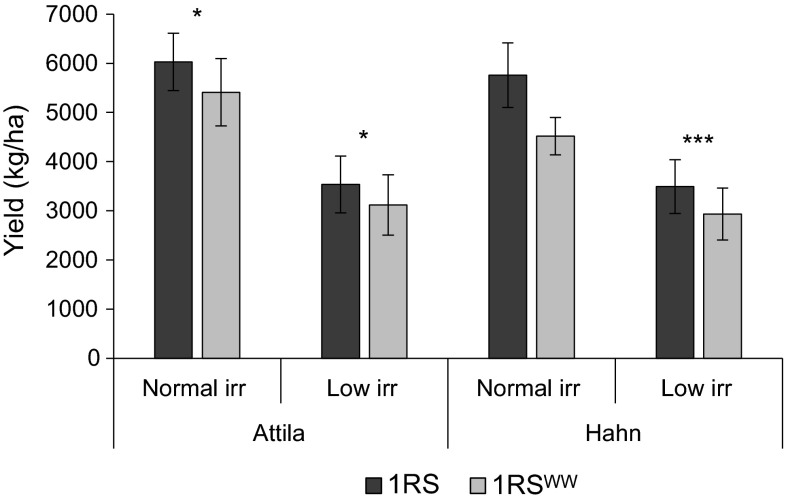
A comparison of the adjusted mean yields of the 1RS and 1RS^WW^ genotypes in the Hahn and Attila backgrounds under two different irrigation regimes from the DREC 2010–2011 field

The ANCOVA for yield showed significant effects of irrigation treatment (*P* < 0.0001) and genotype (*P* < 0.0001). Similar to previous experiments, the 1RS genotype showed higher grain yield than the 1RS^WW^ genotype. In the low irrigation treatment, the effect of genotype was significant in both the Attila (*P* = 0.0190) and Hahn (*P* = 0.0002) backgrounds (Fig. 5). In the normal irrigation treatment, the effect of genotype was significant only in the Attila background (*P* = 0.0387, Fig. 5), though it was marginally non-significant in the Hahn background (*P* = 0.07). Large error bars in Fig. 5 reflect the large amount of variability in the experiment. Overall, results from this experiment agree with those from previous experiments in that the 1RS genotype performs better than the 1RS^WW^ genotype. No significant interactions were detected in this experiment, likely because of the larger variability.

### Mapping of the region affecting grain yield, canopy water status and CID

The experiments described above consistently showed that the introgression of the proximal and distal wheat chromosome segments into the 1RS chromosome arm resulted in a significant reduction in grain yield, canopy water status and CID. To better characterize the influence of these two regions on these differences, we generated two additional NILs in the relatively more sensitive Hahn background, one carrying only the wheat proximal region (Hahn 1RS^WR^) and the other carrying only the wheat distal region (Hahn 1RS^RW^) (see Fig. 1) and evaluated them in field experiments similar to those described above.

#### Davis 2011–2012 preliminary CSR results for mapping

A limited amount of seed of the Hahn 1RS^WR^ and 1RS^RW^ NILs was available for the 2011–2012 growing season, so a single experiment using 1 m rows was sown at Davis. Normal and limited irrigation treatments were planned with 10 replications each; however, late season rains eliminated water stress across the trial and the 20 blocks were ultimately analyzed together as a single RCBD.

**Fig. 6:**
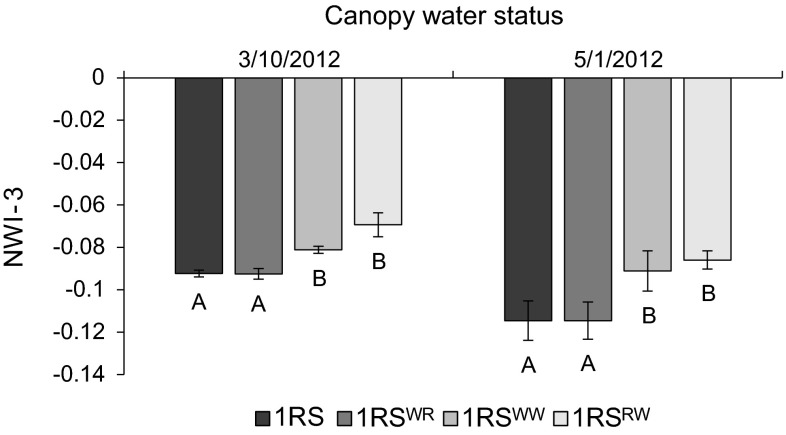
A comparison of the adjusted mean NWI-3 values among the four different NILs in the Hahn background (*more* *negative values* indicate better canopy water status) as measured on March 10, 2012 and May 1, 2012 in Davis. *Different letters* indicate a significant difference between NWI-3 adjusted means for the specified date

The CSR measurements showed that the NILs carrying the original 1RS chromosome arm had significantly more negative NWI-3 values than the respective NILs carrying the 1RS^WW^ chromosome arm, suggesting that this experiment provided appropriate conditions for the discrimination of the different genotypes. In both sets of measurements (taken more than 50 days apart), orthogonal contrasts showed that the lines not carrying the distal wheat segment (Hahn 1RS and Hahn 1RS^WR^) had significantly more negative NWI-3 values than the lines carrying the distal wheat segment (Hahn 1RS^RW^ and Hahn 1RS^WW^) (*P* < 0.0001, both dates, Fig. 6). No significant differences were observed between the NILs within each of these two classes. This preliminary experiment suggested that the gene(s) responsible for improved canopy water status are located in the 1RS segment replaced by the distal wheat introgression.

#### Davis 2012–2013 mapping experiment

**Fig. 7:**
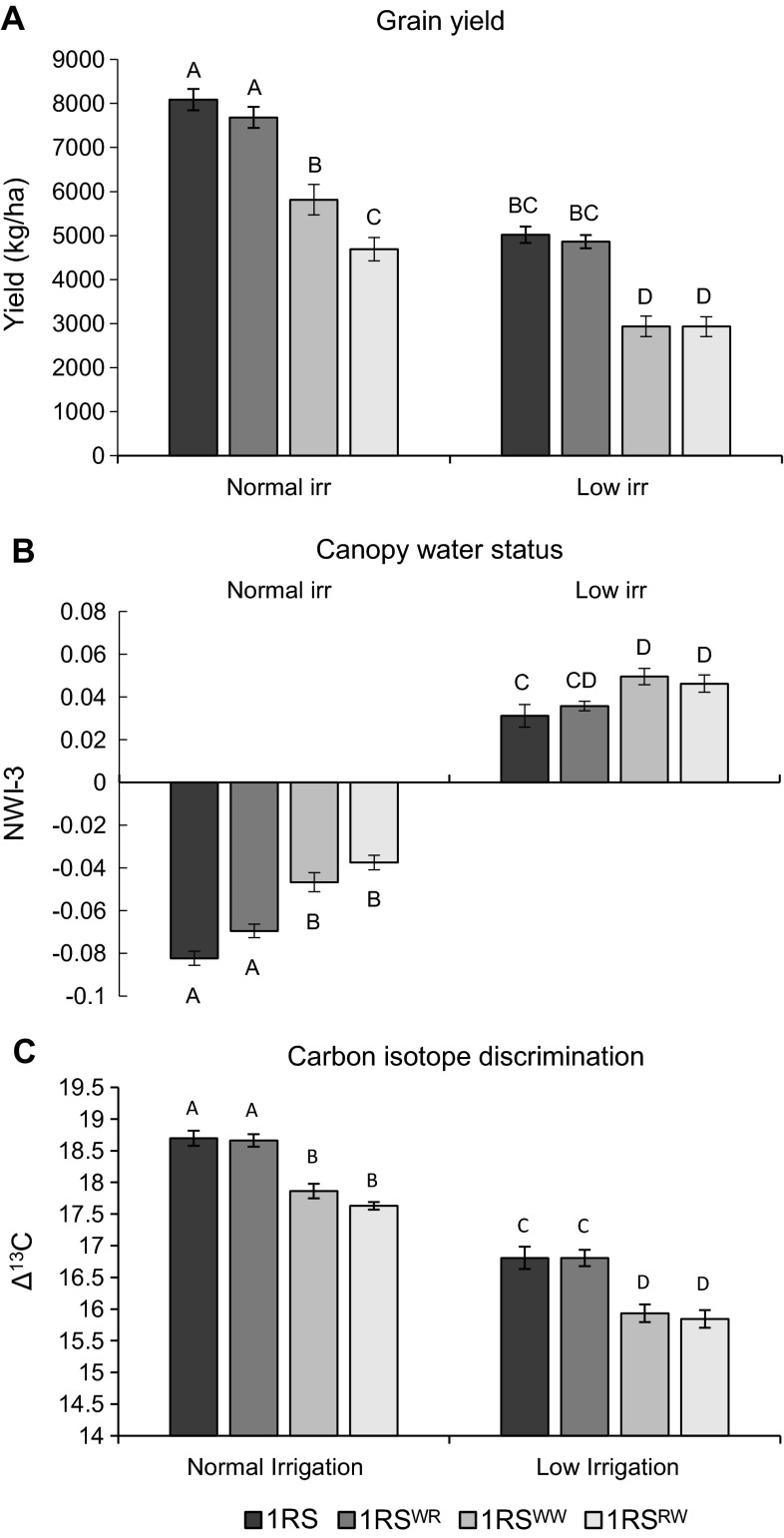
A comparison of the adjusted mean yields, NWI-3 and CID values among 1RS, 1RS^WR^, 1RS^WW^ and 1RS^RW^ genotypes in the Hahn background under two different irrigation regimes (Davis 2012–2013). *Different letters* indicate significant differences between means. **a** Adjusted mean plot yield. **b** Adjusted mean canopy water status estimated by NWI-3 on May 2, 2013 (*more negative values* indicate better canopy water status). **c** Adjusted mean carbon isotope discrimination values measured on mature grains

Using the seed increased in the previous experiment, the four Hahn NILs were evaluated in both normal irrigation and limited terminal irrigation treatments in Davis in 2012–2103 using an RCBD with 10 replications. In both treatments, orthogonal contrasts showed that the genotypes not carrying the distal wheat segment (1RS and 1RS^WR^) had significantly higher grain yield than the genotypes carrying the distal wheat segment (1RS^WW^ and 1RS^RW^) (*P* < 0.0001, Fig. 7a). The 1RS and 1RS^WR^ NILs showed almost identical yields in both treatments, but Tukey’s test showed the 1RS^RW^ genotype had significantly lower grain yield than the 1RS^WW^ genotype in the irrigated treatment (Fig. 7a). In this experiment, there was a significant genotype by irrigation treatment interaction (*P* = 0.0019).

The CSR results showed a similar trend to the 2011–2012 preliminary CSR data and were consistent with the grain yield data described above. A significant genotype by irrigation treatment interaction was detected for the CSR data (*P* = 0.0002). Orthogonal contrasts showed that the lines not carrying the distal wheat segment (1RS and 1RS^WR^) had more negative NWI-3 values than the lines carrying the distal wheat segment (1RS^WW^ and 1RS^RW^) in both normal (*P* = 0.0004) and low irrigation (P < 0.0001) treatments. However, a more conservative Tukey’s test failed to detect significant differences between the 1RS^WR^ and 1RS^RW^ NILs in the low irrigation treatment (Fig. 7b).

The CID data showed significant effects of both genotype (*P* < 0.0001) and treatment (*P* < 0.0001). Unlike the grain yield and CSR results, the genotype by treatment interaction was non-significant (*P* = 0.88). The CID data showed a clear separation of the lines not carrying the distal wheat segment (1RS and 1RS^WR^) and the lines carrying the distal wheat segment (1RS^WW^ and 1RS^WW^), both using a contrast between these two groups (*P* < 0.0001) and in pairwise mean comparisons using Tukey’s test (Fig. 7c).

**Fig. 8:**
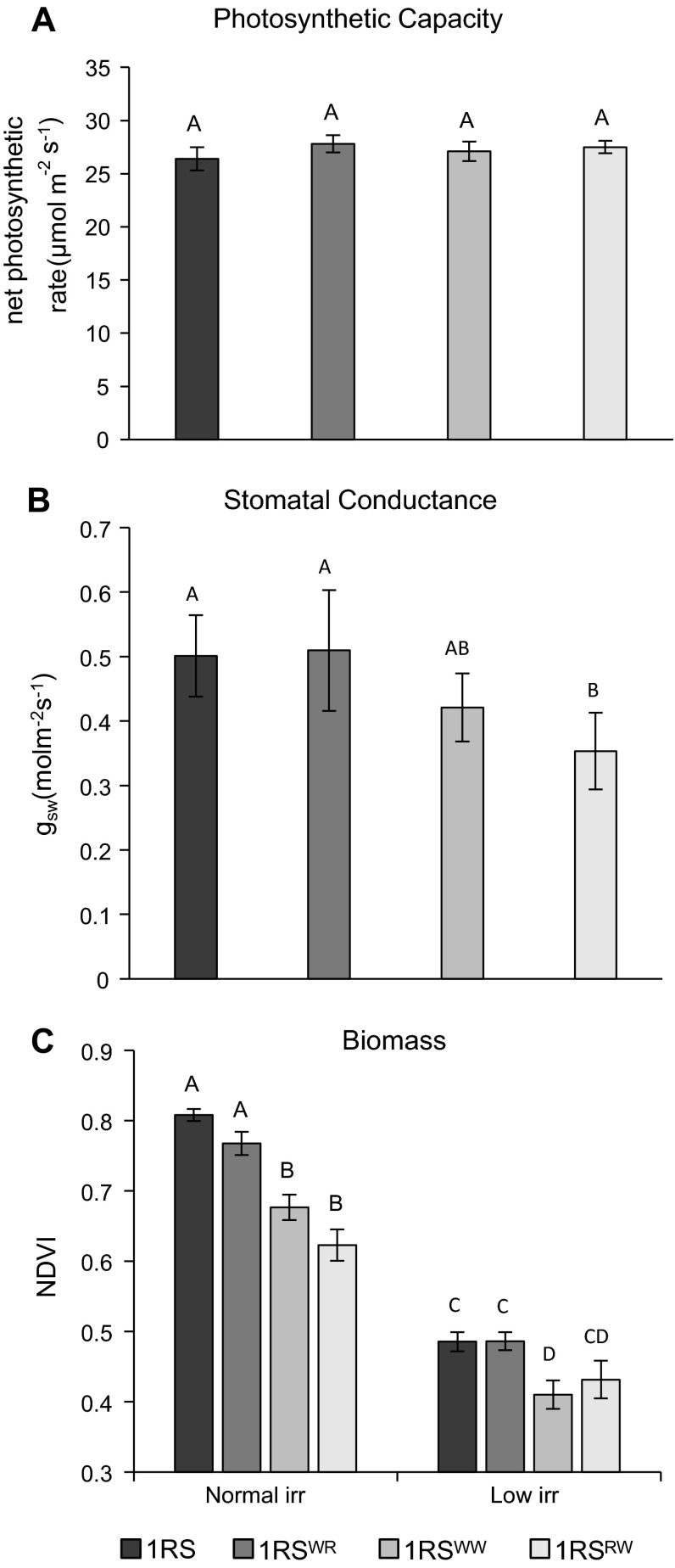
A comparison of the adjusted mean photosynthetic capacity, stomatal conductance and NDVI values among 1RS, 1RS^WR^, 1RS^WW^ and 1RS^RW^ genotypes in the Hahn background (Davis 2012–2013). *Different letters* indicate significant differences between means. **a** Photosynthetic capacity (photosynthetic rate adjusted for differences in stomatal conductance) measured on April 11, 2013, normal irrigation treatment. **b** Stomatal conductance measured on April 11, 2013, normal irrigation treatment. **c** Biomass estimated by CSR using NDVI (*higher values* indicate more green biomass) measured on May 2, 2013, normal and low irrigation treatments

Gas exchange measurements made on April 11 in the irrigated treatment showed a significant effect of genotype on stomatal conductance (*P* = 0.0106). Orthogonal contrasts among adjusted means showed that the lines not carrying the distal wheat segment (1RS and 1RS^WR^) had 29.2 % higher (*P* = 0.0015) stomatal conductance than the lines carrying the distal wheat segment (1RS^WW^ and 1RS^RW^) (Fig. 8a). There were no significant differences in net photosynthetic rates among the different genotypes.

Individual leaf CID Δ^13^C values predicted based on intercellular CO_2_ measurements showed a significant correlation with the actual CID values measured using the mature grain (*R* = 0.57, *P* = 0.0001). When the correlation was calculated using the means of the four different genotypes, the correlation was even higher (*R* = 0.97, *P* = 0.0291). This high correlation between predicted and observed CID values suggests that even though the gas exchange data were evaluated in a single day on a single leaf, they reflect well the accumulated water stress captured by CID.

Finally, for the irrigated treatment in this experiment, we analyzed canopy coverage by estimating green biomass using the CSR NDVI (taken May 2). These measurements showed that the lines not carrying the distal wheat segment (1RS and 1RS^WR^) had significantly higher canopy coverage than the lines carrying the distal introgressed wheat segment (1RS^WW^ and 1RS^RW^), both in the normal (21.2 % higher, contrast *P* < 0.0001) and limited (11.5 %, contrast *P* < 0.0001) irrigation treatments (Fig. 8c).

### Characterization of the distal 1RS region

The localization of the improved field performance of the distal region of the 1RS chromosome arm motivated a more detailed characterization of this region. From the 103 lines with 1RS/1BS recombination events identified previously (Lukaszewski 2000), we selected seven with the most distal recombination events (between the telomere and *Pm8*). These seven lines include 1B + 40 and T-9, which were the lines used to generate the distal wheat introgression (Lukaszewski 2000) (Table 3). Characterization of these lines with seven molecular markers and the cytogenetic telomeric marker revealed the existence of two additional recombination events in 1B + 40, creating a small interstitial rye segment that was not detected previously (Table 3). The presence of this interstitial rye region suggests that recombination between 1B + 40 and T-9 could have occurred either in the wheat region, as previously assumed, or in the short interstitial rye region shared by the two lines. Assuming a total short arm length of 50 cM, we estimated the genetic distance between the telomere and *Pm8* to be approximately 3.9 cM.

**Table 3:** Genetic characterization of seven lines with 1BS/1RS recombination events in the distal region of the short arm (W = wheat allele, R = rye allele)

Marker	Line							
	1RS^RW^	T-9	T-21	1B + 14	1B + 48	1B + 42	1B + 32	1B + 40
Telomere^a^	R	W	W	R	R	R	R	R
*gpw7059* ^b^	W	W	W	R	R	R	R	W
*Gli*-*B1/Glu*-*B3* (*psp3000*)^c^	W	W	W	R	R	R	R	W
*IB*-*267* ^d^	R	W	W	R	R	R	R	R
*iag95* ^d^	R	R	R	W	R	R	R	R
*tsm120* ^e^	R	R	R	W	W	W	W	R
*Pm8* (*sfr43*)^f^	R	R	R	W	W	W	W	R
*Ucr8* ^g^	R	R	R	W	W	W	W	W

The names of the recombinant lines follow the nomenclature from Lukaszewski (2000)

^a^ Lukaszewski (2000)

^b^ Sourdille and Bernard (unpublished data, http://wheat.pw.usda.gov/cgi-bin/graingenes/report.cgi?class=marker&name=GPW7059)

^c^ Devos et al. (1995)

^d^ Mago et al. (2002)

^e^ Kofler et al. (2008)

^f^ Additional evaluations revealed that lines 1B + 42 and 1B + 32 are susceptible to powdery mildew and were misclassified in the initial study (Lukaszewski 2000). This result is supported by the new molecular marker *sfr43* for *Pm8* (Hurni et al. 2013). Line 1B + 40 was also shown to be positive for *Pm8* using the *sfr43* marker

^g^ Sharma et al. (2009)

Using the eight recombination events shown in Table 3, the molecular markers were arranged in their most likely order, which agrees with previous maps generated for this region (Mago et al. 2002; Kofler et al. 2008). In the 1RS^RW^ line, only *Glu*-*B3/Gli*-*B1* (*psp3000*) and *gpw7059* were mapped within the distal wheat introgression (the relative order of these two markers within this region is not known). With the available recombination events, it is not possible to determine if marker *IB*-*267* is located proximal or distal to the distal wheat introgression. In a map of Imperial rye, Mago et al. (2002) mapped *IB*-*267* proximal to *Glu*-*B3/Gli*-*B1,* so we favored this position in Table 3.

The position of *IB*-*267* also affects the estimate of the length of the introgressed wheat segment. Assuming that the *IB*-*267* position proximal to the *Glu*-*B3/Gli*-*B1* loci observed in Imperial rye (Mago et al. 2002) is conserved in Petkus rye, the interstitial wheat segment can be delimited by the two interstitial recombination events in 1B + 40 with an estimated length of less than 1 cM. However, if we place *IB*-*267* distal to the region, the wheat interstitial segment is delimited by five recombination events and its length is estimated to be less than 2.4 cM. Under both scenarios, the length of the introgressed wheat segment is relatively small.

## Discussion

### Comparison of 1RS and 1R^WW^ effects on grain yield and water status

The genotyping results confirmed that the 1RS and 1RS^WW^ lines were highly isogenic, especially in the Hahn background where the phenotypic differences were more pronounced. These results indicate that the observed differences in grain yield, canopy water status, and CID between the 1RS and 1RS^WW^ NILs are most likely due to the presence or absence of the two introgressed wheat segments.

In most years, locations, treatments, and cultivars, the lines with the original 1RS translocation had significantly higher grain yield than the lines containing the engineered 1RS^WW^ arm. These differences were detected not only in the first 2 years where the 1RS and 1RS^WW^ genotypes were the only genotypes grown (2008–2010), but also in the following 2 years (2011–2013) when additional NILs carrying single wheat segments were also tested. The average grain yield of lines with the 1RS chromosome arm across cultivars and years in all our experiments was 17.9 % higher than the yield of the NILs with the 1RS^WW^ chromosome arm under normal irrigation conditions (including DREC 2010), and 26.9 % higher under low irrigation conditions (including Davis 2009).

All of the trials which included both the Hahn and Attila NILs showed a significant interaction between cultivar and 1RS/1RS^WW^ genotype, with the exception of DREC 2010–2011, likely due to the higher variability in that experiment. Additional statistical analyses of the trials showed that the interaction between genotype and cultivar was determined by differences between the Hahn and Attila lines carrying the 1RS^WW^ genotypes and not between the original Hahn and Attila lines carrying the original 1RS chromosome arm (Online Resource Table S1). This result suggests that the beneficial effect of the 1RS chromosome arm is modulated by the genetic background and that breeders should expect differences in the magnitude of the beneficial effects of the 1RS arm across different breeding lines.

A significant interaction between irrigation treatment and genotype was detected for NWI-3 in Davis 2010–2011 and 2012–2103 and for grain yield in Davis 2012–2013. In all these cases, the differences were larger in the treatment with limited irrigation, confirming that the 1RS chromosome arm is a particularly valuable in stressed environments (Villareal et al. 1991; Carver and Rayburn 1994; Schlegel and Meinel 1994; Moreno-Sevilla et al. 1995; Zarco-Hernandez et al. 2005; Hoffmann 2008; Ehdaie et al. 2011). The association of the 1RS chromatin with higher grain yield under water stress is further reinforced by the significant improvements in canopy water status and higher stomatal conductance observed in the lines with the original 1RS arm compared with those carrying the 1RS^WW^ chromosome arm.

A valuable characteristic of the 1RS translocation for breeding applications is that the beneficial effects of this translocation under stress conditions do not appear to be associated with detrimental effects in environments receiving the normal irrigation treatment. On the contrary, significant improvements in grain yield, NWI-3, CID and stomatal conductance were also detected in the experiments that received normal irrigation.

Days to heading (DTH) was recorded in the Davis 2008–2009 and DREC 2010–2011 experiments and the difference in DTH between the 1RS and 1RS^WW^ genotypes was small (0.58 days) and non-significant (*P* = 0.0735). Although DTH was not recorded in other experiments, in all cases, we observed synchronized heading among genotypes within irrigation treatments.

### Effects of individual wheat segments on grain yield and water status

Our study shows that the replacement of the two original 1RS rye segments with wheat segments to improve bread-making quality (Lukaszewski 2000) is associated with a significant decrease in grain yield and in canopy water status. Although this result indicates that the engineered 1RS^WW^ chromosome may have limited value in direct breeding applications, it also implicates the two rye regions replaced by wheat chromatin as the likely location of the beneficial 1RS gene(s). By separating the two introgressed wheat regions into two independent 1RS.1BL chromosomes (1RS^WR^ and 1RS^RW^), we were able to show that the genetic element(s) responsible for higher yield, improved water status, and higher CID are located in the segment of the 1RS chromosome arm replaced by the distal wheat segment. This result is consistent with one QTL for root traits identified in greenhouse studies (Sharma et al. 2011).

The molecular characterization of the lines with the most distal recombination events showed that the introgressed wheat segment is less than 2.4 cM, and likely less than 1 cM if the inferred position of *IB*-*267* from Mago et al. (2002) is correct. Cytogenetic studies in wheat have shown that the frequency of recombination decreases exponentially with increased distance from the telomere, resulting in smaller physical distances in distal chromosome regions than in proximal regions for a given genetic distance (Dvorak et al. 1984, 1998; Kota et al. 1993; Lukaszewski and Curtis 1993; Hohmann et al. 1994; Delaney et al. 1995a; Mickelson-Young et al. 1995; Delaney et al. 1995b; Gill et al. 1996 ). The fact that only one recombination event out of 103 recombinant lines was detected between the telomere and the *Glu*-*B3/Gli*-*B1* locus (1B + 40) indicates that the introgressed wheat region is close to the telomere. Although this distal location predicts a favorable genetic-to-physical distance ratio, we cannot rule out the possibility of local expansion of repetitive rye sequences in this region. We are currently sequencing the 1RS and 1RS^RW^ chromosome arms to test this hypothesis and to identify potential candidate genes for the differences in grain yield, NWI-3, and stomatal conductance observed between the corresponding NILs.

The multiple phenotypic effects associated with the introgression of the distal wheat segment can be explained by multiple linked genes controlling different traits or by a single gene with pleiotropic effects. Based on the small size of the introgressed wheat segment and our experience in previous positional cloning of genes with pleiotropic effects (Yan et al. 2003, 2004, 2006; Uauy et al. 2006), we favor the hypothesis of a single gene with pleiotropic effects.

### Physiological characterization of lines

Although the main objective of this study was the confirmation and mapping of the beneficial effect of the 1RS segment on yield and canopy water status, we also attempted a preliminary physiological characterization of these effects. In all field trials where there were significant yield differences, there were also significant differences in plant water status, as measured by NWI-3. Although the differences in NWI-3 were larger in the limited irrigation treatment than in the normal irrigation in some experiments, the differences were significant in both. These results show that even under normal irrigation conditions, lines not carrying the distal wheat segment exhibited better water status than lines carrying the distal wheat segment. The higher CID observed in the lines not carrying the distal wheat segment suggested that these lines had their stomata opened for a longer periods than the NILs with the distal wheat segment, a conclusion supported by the stomatal conductance and photosynthetic capacity measurements taken in Davis in 2013.

Carbon isotope discrimination is determined by both stomatal conductance (more open stomata lead to higher discrimination) and photosynthetic rate (a higher rate leads to lower discrimination). Therefore, we measured both traits to determine which of them was responsible for the observed differences in CID. Gas exchange measurements in the normal irrigation treatment showed significantly higher stomatal conductance of the flag leaves of the lines not carrying the distal wheat segment relative to those with carrying distal wheat segment (Fig. 8a). Since no significant differences were detected for photosynthetic capacity (Fig. 8b), we conclude that the differences in CID were caused by differences in stomatal conductance. Taken together, these results suggest that the lines not carrying the distal wheat segment may have increased access to stored soil moisture and achieve higher yields, greater canopy closure, and better water status through increased access to water throughout the season, rather than through water conservation. Plants with a conservative water use strategy often have constrained yield potential in high yielding environments (Condon et al. 1993, 2002; Condon and Richards 1993; López-Castañeda and Richards 1994), which we did not observe in this study in the environments with a normal irrigation regime. One possible explanation for the increased access to soil moisture would be an improved root system (Sharma et al. 2011; Ehdaie et al. 2003).

To summarize the contribution of the different traits to the discrimination among genotypes, we performed a principal component analysis including the different traits measured in the Davis 2012–2013 irrigated field experiment (Online Resource Fig. S2). The first principal component explained 51.4 % of the variation and was the main contributor to the separation of genotypes. Normalized water indexes, CID and carbon isotope ratio measured in flour from mature grains, and the different vegetation coverage indexes showed the highest contributions to the first principal component and to the separation of genotypes. The second principal component explained 14.3 % of the variation and did not contribute to the separation among genotypes. This second component was partially associated with the time of the day when the measurements were taken.

### Future directions and applications

From a practical point of view, the localization of the region associated with increased grain yield and improved canopy water status to the distal region suggests that the 1RS *Sec*-*1* locus associated with sticky dough can be eliminated without losing the positive effect of the 1RS arm on grain yield (Fig. 7a, 1RS^WR^ NIL). However, currently available 1BS/1RS recombination events (Lukaszewski 2000) are insufficient to separate the 1RS distal region with beneficial effects on grain yield from the wheat *Glu*-*B3/Gli*-*B1* loci and their associated beneficial effects on gluten strength. A possible strategy to mitigate the negative impact of the loss of the *Glu*-*B3/Gli*-*B1* loci is to combine the distal 1RS chromosome arm region with the Bx7 over-expressor allele (*Bx7* ^*OE*^) at the *Glu*-*B1* locus in the long arm of chromosome 1B (Ragupathy et al. 2008). This allele carries a duplication of the Bx7 glutenin subunit and is associated with extra strong gluten and dough strength (Radovanovic et al. 2002; Butow et al. 2003; Vawser and Cornish 2004). Recent studies of Argentine wheat cultivars with the 1RS.1BL translocation support the compensatory effect of the *Bx7* ^*OE*^ allele on bread-making quality (Pflüger et al. 2014)

The distal 1RS segment can be incorporated into wheat breeding programs using two different strategies. The first strategy is the use of the 1RS^WR^ chromosome arm described in this study, which has already been tested for its beneficial effects on yield and canopy water status. The disadvantage of this strategy is that almost the complete short arm is blocked from recombination with the homologous wheat 1BS arm, limiting the introgression of favorable wheat alleles present in this region (e.g., stripe rust resistance gene *Yr15*). The second strategy is to introgress only the distal 1RS region using lines 1B + 14, 1B + 48, 1B + 42 or 1B + 32 (Table 3) as donor parents (Table 3). In this configuration, most of the 1BS arm is available for normal recombination and introgression of desirable traits. However, this configuration has not yet been tested for its effects on yield or canopy water status, so we cannot rule out the possibility that the beneficial effects of the distal 1RS segment require the presence of additional rye genes located in other regions of the 1RS arm.

In summary, the precise mapping of the beneficial effects of the 1RS translocation on grain yield and water canopy status opens the door to new strategies to utilize this valuable introgression in wheat breeding programs. It is also a first step in the identification of the gene(s) responsible for these important agronomic traits.

The 1RS^WW^ (PI 672837), 1RS^WR^ (PI 672838) and 1RS^RW^ (PI 672839) NILs in the Hahn background have been deposited in the National Small Grains Collection and are publically available.

#### **Author contributions**

TH and JD directed the project. MB and TH developed isogenic lines. TH, IH, LJ conducted field experiments, MG performed physiological studies, and TH wrote first draft of the manuscript. All authors reviewed the manuscript. JD wrote the grants that supported the project and provided extensive editing to the manuscript.

## Electronic supplementary material

Below is the link to the electronic supplementary material.

Supplementary material 1 (PDF 122 kb)

## References

[CR1] Butow BJ, Ma W, Gale KR, Cornish GB, Rampling L, Larroque O, Morell MK, Békés F (2003). Molecular discrimination of Bx7 alleles demonstrates that a highly expressed high-molecular-weight glutenin allele has a major impact on wheat flour dough strength. Theor Appl Genet.

[CR2] Carver F, Rayburn AL (1994). Comparison of related wheat stocks possessing 1B or 1RS.1BL chromosomes: agronomic performance. Crop Sci.

[CR3] Condon AG, Richards RA, Ehleringer JR, Hall AE, Farquhar GD (1993). Exploiting genetic variation in transpiration efficiency in wheat: an agronomic view. Stable isotope plant carbon-water relations.

[CR4] Condon AG, Richards RA, Farquhar GD (1993). Relationships between carbon isotope discrimination, water use efficiency and transpiration efficiency for dryland wheat. Crop Pasture Sci.

[CR5] Condon AG, Richards RA, Rebetzke GJ, Farquhar GD (2002). Improving intrinsic water-use efficiency and crop yield. Crop Sci.

[CR6] Delaney DE, Nasuda S, Endo TR, Gill BS, Hulbert SH (1995). Cytologically based physical maps of the group-2 chromosomes of wheat. Theor Appl Genet.

[CR7] Delaney DE, Nasuda S, Endo TR, Gill BS, Hulbert SH (1995). Cytologically based physical maps of the group 3 chromosomes of wheat. Theor Appl Genet.

[CR8] Devos KM, Bryan GJ, Collins AJ, Stephenson P, Gale MD (1995). Application of two microsatellite sequences in wheat storage proteins as molecular markers. Theor Appl Genet.

[CR9] Dvorak J, Lassner MW, Kota RS, Chen KC (1984). The distribution of the ribosomal RNA genes in the *Triticum speltoides* and *Elytrigia elongata* genomes. Can J Genet Cytol.

[CR10] Dvorak J, Luo MC, Yang ZL (1998). Restriction fragment length polymorphism and divergence in the genomic regions of high and low recombination in self-fertilizing and cross-fertilizing *Aegilops* species. Genetics.

[CR11] Ehdaie B, Whitkus RW, Waines JG (2003). Root biomass, water-use efficiency, and performance of wheat–rye translocations of chromosomes 1 and 2 in spring bread wheat “Pavon”. Crop Sci.

[CR12] Ehdaie B, Layne AP, Waines JG (2011). Root system plasticity to drought influences grain yield in bread wheat. Euphytica.

[CR13] FAOSTAT (2014) FAOSTAT. http://faostat.fao.org/

[CR14] Farquhar GD, Ehleringer JR, Hubick KT (1989). Carbon isotope discrimination and photosynthesis. Annu Rev Plant Biol.

[CR15] Fenn D, Lukow OM, Bushuk W, Depauw RM (1994). Milling and baking quality of 1BL/1RS translocation wheats. I. Effects of genotype and environment. Cereal Chem.

[CR16] Gilbert ME, Zwieniecki MA, Holbrook NM (2011). Independent variation in photosynthetic capacity and stomatal conductance leads to differences in intrinsic water use efficiency in 11 soybean genotypes before and during mild drought. J Exp Bot.

[CR17] Gill KS, Gill BS, Endo TR, Taylor T (1996). Identification and high-density mapping of gene-rich regions in chromosome group 1 of wheat. Genetics.

[CR18] Graybosch RA (2001). Uneasy unions: quality effects of rye chromatin transfers to wheat. J Cereal Sci.

[CR19] Gutierrez M, Reynolds MP, Raun WR, Stone ML, Klatt AR (2010). Spectral water indices for assessing yield in elite bread wheat genotypes under well-irrigated, water-stressed, and high-temperature conditions. Crop Sci.

[CR20] Hoffmann B (2008). Alteration of drought tolerance of winter wheat caused by translocation of rye chromosome segment 1RS. Cereal Res Commun.

[CR21] Hohmann U, Endo TR, Gill KS, Gill BS (1994). Comparison of genetic and physical maps of group 7 chromosomes from *Triticum aestivum* L. Mol Gen Genet.

[CR22] Hurni S, Brunner S, Buchmann G, Herren G, Jordan T, Krukowski P, Wicker T, Yahiaoui N, Mago R, Keller B (2013). Rye *Pm8* and wheat *Pm3* are orthologous genes and show evolutionary conservation of resistance function against powdery mildew. Plant J.

[CR23] Kim W, Johnson J (2004) Agronomic effect of wheat-rye translocation carrying rye chromatin (1R) from different sources. Crop Sci 1254–1258

[CR24] Kofler R, Bartoš J, Gong L, Stift G, Suchánková P, Šimková H, Berenyi M, Burg K, Doležel J, Lelley (2008) Development of microsatellite markers specific for the short arm of rye (*Secale cereale* L.) chromosome 1. Theor Appl Genet 117:915–926 10.1007/s00122-008-0831-2 18626624

[CR25] Kota RS, Gill KS, Gill BS, Endo TR (1993). A cytogenetically based physical map of chromosome 1B in common wheat. Genome.

[CR26] López-Castañeda C, Richards RA (1994). Variation in temperate cereals in rainfed environments III. Water use and water-use efficiency. Field Crop Res.

[CR27] Lukaszewski AJ (2000). Manipulation of the 1RS.1BL translocation in wheat by induced homoeologous recombination. Crop Sci.

[CR28] Lukaszewski AJ, Curtis CA (1993). Physical distribution of recombination in B-genome chromosomes of tetraploid wheat. Theor Appl Genet.

[CR29] Mago R, Spielmeyer W, Lawrence G, Lagudah E, Ellis J, Pryor A (2002). Identification and mapping of molecular markers linked to rust resistance genes located on chromosome 1RS of rye using wheat-rye translocation lines. Theor Appl Genet.

[CR30] Mickelson-Young L, Endo TR, Gill BS (1995). A cytogenetic ladder-map of the wheat homoeologous group-4 chromosomes. Theor Appl Genet.

[CR31] Moreno-Sevilla B, Baenziger PS, Peterson CJ, Graybosch RA, McVey DV (1995). The 1BL/1RS translocation: agronomic performance of F3-derived lines from a winter wheat cross. Crop Sci.

[CR32] Owuoche JO, Sears RG, Brown-Guedira GL, Gill BS, Fritz AK (2003). Heterotic effects of wheat-rye chromosomal translocations on agronomic traits of hybrid wheat (*Triticum aestivum* L.) under an adequate moisture regime. Euphytica.

[CR33] Peñuelas J, Isla R, Filella I, Araus JL (1997). Visible and near-infrared reflectance assessment of salinity effects on barley. Crop Sci.

[CR34] Pflüger L, Cuniberti M, Babinec F, Helguera M (2014). Evaluation of the effect of the *Glu*-*B1al* allele on bread making quality parameters in Argentine breads.

[CR35] Purnhauser L, Bóna L, Láng L (2010). Occurrence of 1BL.1RS wheat-rye chromosome translocation and of *Sr36/Pm6* resistance gene cluster in wheat cultivars registered in Hungary. Euphytica.

[CR36] Rabinovich SV (1998). Importance of wheat-rye translocations for breeding modern cultivar of *Triticum aestivum* L. Euphytica.

[CR37] Radovanovic N, Cloutier S, Brown D, Humphreys DG, Lukow OM (2002). Genetic variance for gluten strength contributed by high molecular weight glutenin proteins. Cereal Chem.

[CR38] Ragupathy R, Naeem HA, Reimer E, Lukow OM, Sapirstein HD, Cloutier S (2008). Evolutionary origin of the segmental duplication encompassing the wheat *GLU*-*B1* locus encoding the overexpressed Bx7 (Bx7(OE)) high molecular weight glutenin subunit. Theor Appl Genet.

[CR39] Rouse JW, Haas RH, Schell JA, Deering DW (1974). Monitoring vegetation systems in the Great Plains with ERTS. NASA Spec Publ.

[CR40] Schlegel R, Meinel A (1994). A quantitative trait locus (QTL) on chromosome arm 1RS of rye and its effect on yield performance of hexaploid wheat. Cereal Res Commun.

[CR41] Sharma S, Bhat PR, Ehdaie B, Close TJ, Lukaszewski AJ, Waines JG (2009). Integrated genetic map and genetic analysis of a region associated with root traits on the short arm of rye chromosome 1 in bread wheat. Theor Appl Genet.

[CR42] Sharma S, Xu S, Ehdaie B, Hoops A, Close TJ, Lukaszewski AJ, Waines JG (2011). Dissection of QTL effects for root traits using a chromosome arm-specific mapping population in bread wheat. Theor Appl Genet.

[CR43] Shearman VJ, Scott RK, Foulkes MJ (2005). Crop physiology and metabolism. Physiological processes associated with wheat yield progress in the UK.

[CR44] Shimizu Y, Nasuda S, Endo TR (1997). Detection of the *Sec*-*1* locus of rye by a PCR-based method. Genes Genet Syst.

[CR45] Uauy C, Distelfeld A, Fahima T, Blechl A, Dubcovsky J (2006). A NAC gene regulating senescence improves grain protein, zinc and iron content in wheat. Science.

[CR46] Vawser MJ, Cornish GB (2004). Over-expression of HMW glutenin subunit *Glu*-*B1* 7x in hexaploid wheat varieties (*Triticum aestivum*). Aust J Agric Res.

[CR47] Villareal RL, Rajaram S, Mujeeb-kazi A, Del Toro E (1991). The effect of chromosome 1B/1R translocation on the yield potential of certain spring wheats (*Triticum aestivum* L.). Plant Breed.

[CR48] Villareal RL, Banuelos O, Mujeeb-Kazi A, Rajaram S (1998). Agronomic performance of chromosomes 1B and T1BL.1RS near-isolines in the spring bread wheat Seri M82. Euphytica.

[CR49] Wang S, Wong D, Forrest K, Allen A, Chao S, Huang BE, Maccaferri M, Salvi S, Milner SG, Cattivelli L (2014). Characterization of polyploid wheat genomic diversity using a high-density 90,000 single nucleotide polymorphism array. Plant Biotechnol J.

[CR50] Yan L, Loukoianov A, Tranquilli G, Helguera M, Fahima T, Dubcovsky J (2003). Positional cloning of wheat vernalization gene *VRN1*. Proc Natl Acad Sci USA.

[CR51] Yan L, Loukoianov A, Blechl A, Tranquilli G, Ramakrishna W, SanMiguel P, Bennetzen JL, Echenique V, Dubcovsky J (2004). The wheat *VRN2* gene is a flowering repressor down-regulated by vernalization. Science.

[CR52] Yan L, Fu D, Li C, Blechl A, Tranquilli G, Bonafede M, Sanchez A, Valarik M, Yasuda S, Dubcovsky J (2006). The wheat and barley vernalization gene *VRN3* is an orthologue of *FT*. Proc Natl Acad Sci USA.

[CR53] Zarco-Hernandez JA, Santiveri F, Michelena A, Javier Peña R (2005). Durum wheat (*Triticum turgidum*, L.) carrying the 1BL/1RS chromosomal translocation: agronomic performance and quality characteristics under Mediterranean conditions. Eur J Agron.

