# Supplementary material for: Mapping a region within the 1RS.1BL translocation in common wheat affecting grain yield and canopy water status
Source: Theor Appl Genet. 2014 Oct 18;127(12):2695–709. doi: 10.1007/s00122-014-2408-6 (PMC4236633; doi:10.1007/s00122-014-2408-6)
Supplement: Supplementary file 1 — Supplementary material 1 (PDF 122 kb) [file 122_2014_2408_MOESM1_ESM.pdf]

Online Resource Theoretical and Applied Genetics

Mapping a region within the 1RS.1BL translocation in common wheat affecting grain yield and canopy water status

Tyson Howell[1], Iago Hale[1,2], Ljupcho Jankuloski[1, 3, 4], Marcos Bonafede[1,5], Matthew Gilbert[1], and Jorge Dubcovsky[1, 6*]

(1) Department of Plant Sciences, University of California, Davis, CA 95616, USA

(2) Department of Biological Sciences, University of New Hampshire, Durham, NH 03824, USA

(3) Department of Genetics and Plant Breeding, Faculty of Agricultural Sciences and Food, 1000 Skopje, Macedonia

(4) Plant Breeding and Genetics Section, Joint FAO/IAEA Division of Nuclear Techniques in Food and Agriculture, IAEA, 1400 Vienna, Austria

(5) Instituto de Recursos Biológicos, CIRN, Instituto Nacional de Tecnología Agropecuaria (INTA), Buenos Aires, Argentina

(6) Howard Hughes Medical Institute, Chevy Chase, MD 20815, USA

Corresponding author: Jorge Dubcovsky. Email: jdubcovsky@ucdavis.edu, Phone (530) 752-5159

Table S1 Statistical analyses of the effect of genetic background (cv. Hahn vs. cv. Attila) in the presence of both the full 1RS arm and the engineered $1RS^{WW}$ arm. In most experiments, the differences between cultivars in yield, carbon isotope discrimination, (CID) and canopy spectral reflectance were significant only in the presence of the $1RS^{WW}$ genotype

Location	Year	Trait	Irrigation treatment	Cultivar effect 1RS	Cultivar effect $1RS^{WW}$
Davis	08-09	Yield	-	0.5345	<0.0001***
DREC	09-10	Yield	-	0.4024	0.0016**
		CID	-	0.0875	0.0168*
Davis	10-11	Yield	Normal	0.9009	0.0412*
			Low	0.3690	0.0048**
		CID	Normal	0.7608	<0.0001***
			Low	0.1989	<0.0001***
		NWI-3	Normal	0.5510	0.0006***
			Low	0.8429	<0.0001***
DREC	10-11	Yield	Normal	0.0844	0.0574
			Low	0.7165	0.4129

* = <0.05, ** = <0.01, *** = <0.001.

Fig. S1 Field “heat map” of yield for the DREC 2010-2011 trial. H= Hahn plots (in brown), A= Attila plots (in blue). 1RS genotypes are underlined and 1RS^{WW} genotypes are not underlined to facilitate comparisons between genotypes . Background color indicates yield level, with high yield in green, low yield in red and intermediate yield in yellow. Lines indicate blocks. A) Normal irrigation section. B) Low irrigation section. The observed decrease in yield with increased distance to the irrigation alley was corrected using this distance as a covariable (see main text).

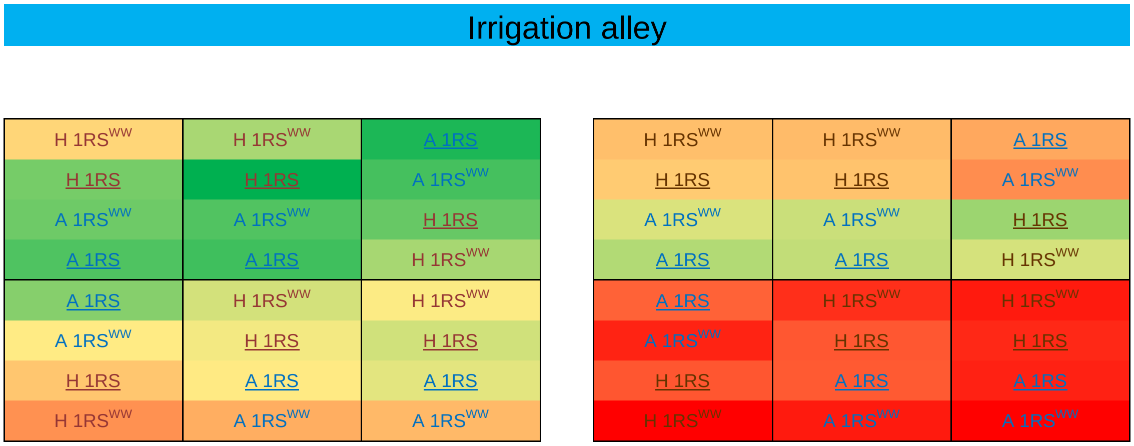

A) Normal Irrigation treatment

B) Low irrigation treatment

Fig. S2 Principle component biplot integrating different traits recorded in the Davis 2012-2013 field experiment. The lengths of the vectors for each trait are proportional to their contribution to the different components. Variables are abbreviated as follows: **CID** - carbon isotope discrimination, measured in flour from mature grains; **CIDpred** - predicted carbon isotope discrimination; **CIR** - carbon isotope ratio, measured in flour from mature grains; **CIRpred** - predicted carbon isotope ratio; **Cond** - stomatal conductance; **CoverByEye** - canopy closure, estimated by eye; **DW_880** - downwelling intensity of 880 nm light; **DW_970** - downwelling intensity of 970 nm light; **DW_NWI3** - NWI3 index calculated from DW_970 and DW_880; **GNDVI** - green normalized vegetation index (Babar et al. 2006); **JazTime and DW_time** - measurement time for CSR indices; **NDVI** - normalized vegetation index; **NWI1** - normalized water index 1 (Babar et al. 2006); **NWI2** - normalized water index 2 (Babar et al. 2006); **NWI3** - normalized water index 3 (Gutierrez et al. 2010); **NWI4** - normalized water index 4 (Gutierrez et al. 2010); **Photo** - photosynthetic rate; **PRI** - photochemical reflective index (Babar et al. 2006); **RNDVI** - red normalized vegetation index (Babar et al. 2006); **SPAD** - relative chlorophyll content; **SR** - simple ratio (Babar et al. 2006); **VPDair** - vapor pressure deficit of air. Ellipses for each group are drawn at the default 68% confidence level for the ggbiplot package in R.

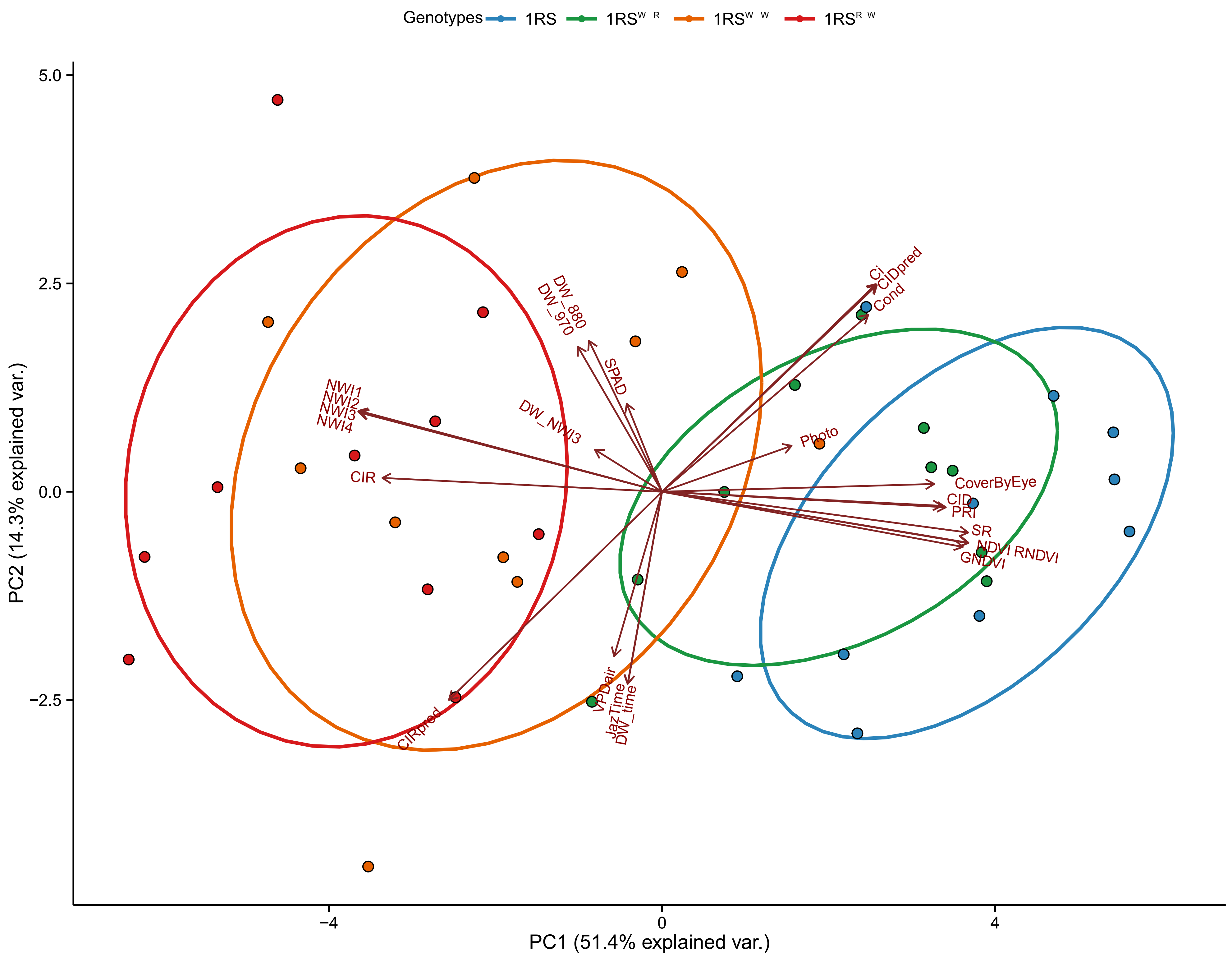

Genotypes
1RS
1RSW R
1RSW W
1RSR W
5.0
2.5
0.0
−2.5
PC2 (14.3% explained var.)
−4
0
4
PC1 (51.4% explained var.)
Ci
CIDpred
Cond
DW_880
DW_970
SPAD
NWI1
NWI2
NWI3
NWI4
DW_NWI3
Photo
CIR
CoverByEye
CID
PRI
SR
NDVI
RNDVI
GNDVI
VPDair
JazTime
DW_time
CIRpred

REFERENCES ONLINE RESOURCE

Babar MA, Reynolds MP, Van Ginkel M, Klatt AR, Raun WR, Stone M L (2006). Spectral reflectance indices as a potential indirect selection criteria for wheat yield under irrigation. Crop Science, 46(2), 578-588.
